# Transcription factor ELF4 in physiology and diseases: Molecular roles and clinical implications

**DOI:** 10.1016/j.gendis.2024.101394

**Published:** 2024-08-23

**Authors:** Dian Hu, Zerui Zhang, Yijun Wang, Siwen Li, Jiaqian Zhang, Zhangfan Wu, Mengyu Sun, Junqing Jiang, Danfei Liu, Xiaoyu Ji, Shuai Wang, Yufei Wang, Xiangyuan Luo, Wenjie Huang, Limin Xia

**Affiliations:** a Department of Gastroenterology, Institute of Liver and Gastrointestinal Diseases, Hubei Key Laboratory of Hepato-Pancreato-Biliary Diseases, Tongji Hospital of Tongji Medical College, Huazhong University of Science and Technology, Wuhan, Hubei 430030, China; b Hubei Key Laboratory of Hepato-Pancreato-Biliary Diseases, Hepatic Surgery Center, Tongji Hospital, Tongji Medical College, Huazhong University of Science and Technology, Clinical Medicine Research Center for Hepatic Surgery of Hubei Province, Key Laboratory of Organ Transplantation, Ministry of Education and Ministry of Public Health, Wuhan, Hubei 430030, China; c Key Laboratory of Integrated Oncology and Intelligent Medicine of Zhejiang Province, Department of Hepatobiliary and Pancreatic Surgery, Affiliated Hangzhou First People's Hospital, Westlake University School of Medicine, Hangzhou, Zhejiang 310006, China; d State Key Laboratory of Holistic Integrative Management of Gastrointestinal Cancers and National Clinical Research Center for Digestive Diseases, Xijing Hospital of Digestive Diseases, Fourth Military Medical University, Xi'an, Shannxi 710032, China

**Keywords:** Biomarker, Cancer, ELF4/MEF, Immune system, Metastasis

## Abstract

Transcription factor E74 like ETS transcription factor 4 (ELF4), a member of the ETS family, is highly expressed in normal human hematopoietic tissue, ovary, placenta, colon, and certain pathological cell lines. During normal physiological processes, ELF4 regulates differentiation in osteogenic, adipocyte, and neuronal types. It also exerts a critical impact on the development of the immune system. However, its function is dysregulated through posttranslational modifications, gene fusions, and complex signaling crosstalk under pathological conditions. Furthermore, serving as a double-edged sword in cancer, ELF4 exhibits both tumor-suppressing and tumor-promoting effects. Specifically, ELF4 plays a critical role in cancer metastasis, proliferation, and modulation of the tumor microenvironment. This review provides an in-depth overview of the molecular structure and post-translational modifications of ELF4. It also summarizes the hallmarks of ELF4 in physiology and diseases, with a particular focus on its significance in oncology. Notably, this review underscores the potential of ELF4 as a prognostic biomarker, highlighting its clinical relevance. Finally, it discusses unresolved questions and future research directions of ELF4. An in-depth understanding of ELF4 biology could facilitate its clinical translation and offer promising targeted therapeutic strategies.

## Introduction

The E26 transformation-specific (ETS) gene originally comes from the E twenty-six oncogene in the avian erythroblastosis virus E26.^1^^,^^2^ ETS protein plays a critical role in regulating downstream targets as a transcription factor. The ETS family, comprising 28 transcription factors, features a structurally conserved ETS DNA binding domain characterized by 3 α-helices and 4 β-sheets.^3^^,^^4^ The functional region is the residues in the third α-helix, which resides in the wing between the third and fourth β-sheet, and the loop between the second and third α-helix. ETS factors are precisely regulated by coregulatory proteins, microRNAs, and numerous posttranslational modifications.^5^, ^6^, ^7^ Additionally, ETS factors are demonstrated to transcriptionally regulate key molecules of multiple signaling pathways including mitogen-activated protein kinase (MAPK) pathway, nuclear factor kappa-B (NF-κB) pathway, and transforming growth factor-β (TGF-β) signaling.^8^, ^9^, ^10^, ^11^, ^12^ Functionally, a review from our laboratory has summarized that ETS factors are involved in multiple physiological processes including cell cycle control, proliferation, differentiation, apoptosis, hematopoiesis, and angiogenesis.^13^ Not surprisingly, their dysregulation contributes to the occurrence of diseases and even cancer.

As a new member of the ETS family, E74 like ETS transcription factor 4 (ELF4) was initially isolated from a human megakaryocytic cell line. ELF4 protein, also known as myeloid elf-1-like factor (MEF), has a central ETS domain.^14^ ELF4 also belongs to the ELF1/E74 subfamily and binds to the DNA-binding sequence (-WGGA-) similarly to ELF1. Thus, ELF4 regulates the expression of granulocyte-macrophage colony-stimulating factor (GM-CSF) and interleukin-2 (IL-2) known as ELF1 targets.^14^ ELF4 is constitutively localized in the nucleus and is highly expressed in normal human hematopoietic tissue, non-hematopoietic tissue (ovary, placenta, and colon), and myeloid leukemia cell lines.^14^ The expression of ELF4 is tightly controlled by post-translational modifications and other signaling pathways.^15^, ^16^, ^17^ ELF4 plays a regulatory role in physiological processes, particularly the development of the immune system.^18^ In past years, it has been demonstrated that ELF4 acts as both a tumor suppressor and an activator in a cell-context manner.^19^^,^^20^ Furthermore, ELF4 exerts an essential impact on the malignant properties of tumors including invasion, metastasis, and proliferation.^19^^,^^21^^,^^22^ In some tumors, including colorectal cancer, ELF4 expression is elevated compared with normal tissues and is associated with poor prognosis.^22^^,^^23^

This review illustrates the molecular architecture, post-translational modifications, transcriptional regulation, and multifaceted physiological functions of ELF4. Dysregulation of ELF4 contributes to the occurrence of diseases including cancer. This review synopsizes the oncogenic characteristics of ELF4, as a pivotal regulator of both tumor suppression and activation, with a particular emphasis on its roles in tumor invasion, metastasis, proliferation, and immunity. The association of ELF4 with immune cell infiltration is analyzed, as inferred from public databases. Finally, ELF4 is proposed as a potential prognosis biomarker and therapeutic target. A systematic and comprehensive review will enhance the understanding of ELF4 and accelerate the progress of ELF4 from basic research into the clinic.

## Structure

The ELF1/E74 subfamily of the ETS family comprises three transcription factors, ELF1, ELF2, and ELF4. In 1992, ELF1 was initially discovered using a probe from the DNA binding domain of ETS-1.^24^ ELF1, E74-like factor 1, contains a DNA binding domain, which is extremely similar to that of E74. All ETS family members, including ETS-1 and ELF1, contain conserved minimal DNA binding domains (adjacent basic and putative α-helical regions). However, ELF1 and ETS-1 exhibit different DNA binding specificities.^25^ While ETS-1 binds to the GGAA or GGAT core DNA sequence, ELF1 selectively binds to GGAA core sequence. The selectivity may be due to the transformation from lysine residue of conserved region III (CRIII) in ETS-1 to threonine at the corresponding site in ELF1.^26^ In 1996, NERF (new ETS-related factor) was cloned from human spleen, fetal liver, and brain firstly. NERF has high homology with ELF1 in DNA binding sequence by comparing amino acid sequences. Hence, NERF is also named as E74-like factor 2 (ELF2).^27^ Unlike ELF1, ELF2 contains a rhombotin-2 binding domain.^28^, ^29^, ^30^, ^31^

**Figure 1:**
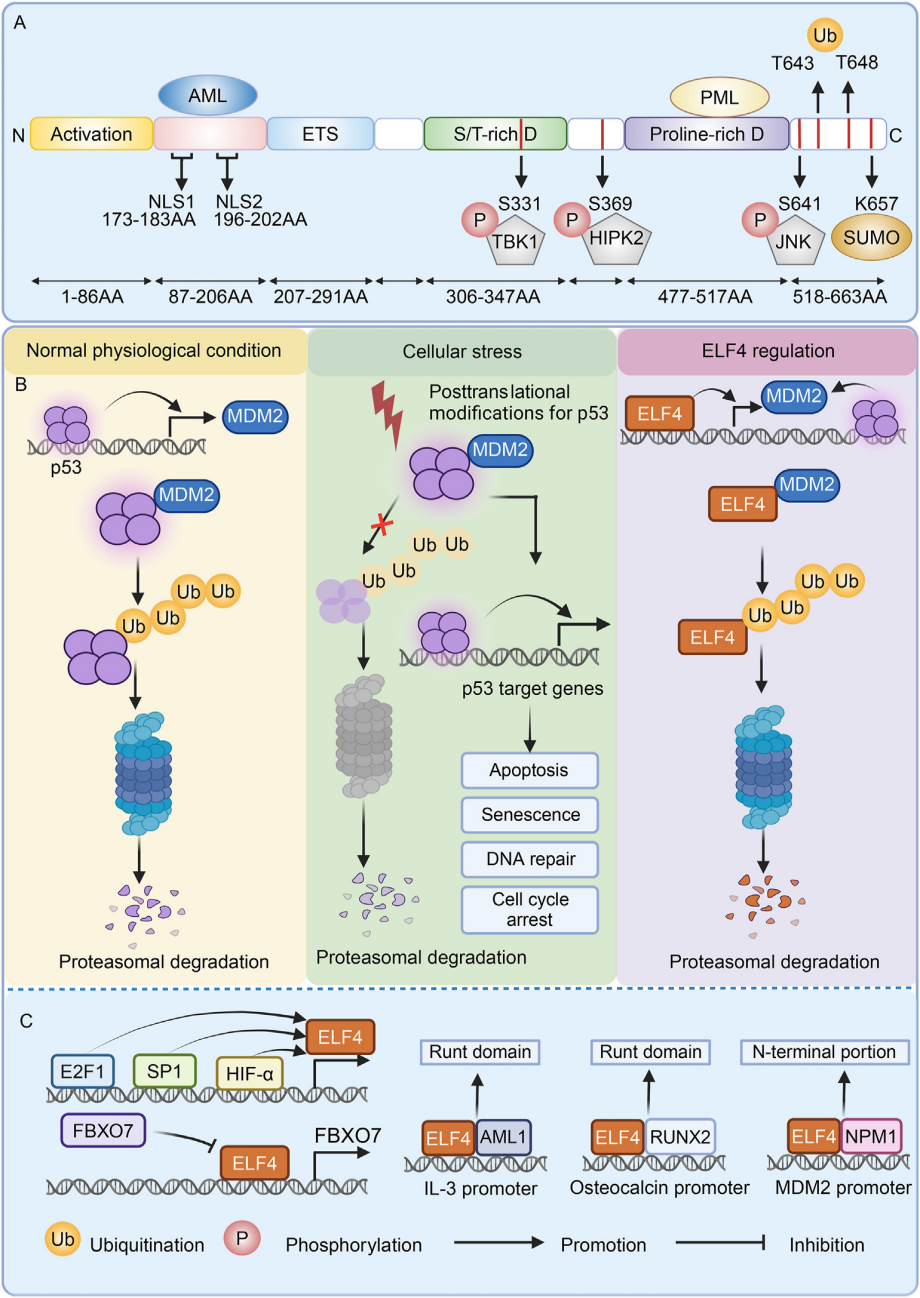
Domain structure and transcriptional regulation of ELF4. **(A)** ELF4 includes six functional domains: an acidic domain, an AML1 interaction domain, a conserved ETS domain, a serine/threonine-rich domain, a proline-rich domain, and two nuclear location signals (NLSs). ELF4 is modified by phosphorylation, ubiquitination, and SUMOylation. The sites of post-translational modification and protein–protein interaction are identified. **(B)** P53/MDM2 axis and ELF4/MDM2 axis generate an autoregulatory negative feedback loop. **(C)** The upstream transcriptional regulation and co-regulator of ELF4 are shown. ELF4, E74 like ETS transcription factor 4; MDM2, mouse double minute 2 homolog; FBXO7, F-box protein 7; NPM1, nucleophosmin 1; HIF-1α, hypoxia inducible factor 1 subunit alpha; E2F1, E2F transcription factor 1; TBK1, TANK-binding kinase 1; HIPK2, homeodomain-interacting protein kinase 2; JNK, c-Jun N-terminal kinase; SUMO, small ubiquitin-like modifier.

The gene encoding ELF4 is located on chromosome X in the region q26.1 and has eight coding exons and one non-coding exon.^14^^,^^32^ ELF4 protein contains 663 amino acids and features six functional domains: an acidic domain, an AML1 interaction domain, a conserved ETS domain, a serine/threonine-rich domain, a proline-rich domain, and two nuclear location signals (NLSs) (Fig. 1A).^14^^,^^33^ As a feature of the ETS family, the conserved ETS domain has 85 amino acids which form a winged helix-turn-helix topology and preferentially binds to purine-rich DNA with a 5′-GGAA/T-3′ core.^4^ Except for the interaction between protein and DNA, the ETS domain is also involved in protein–protein interactions, which mediates various roles including development and oncogenesis.^34^^,^^35^ ELF4 contains two putative NLSs, NLS1 (173–183) and NLS2 (196–202), which are responsible for its nuclear localization (Fig. 1A).^33^^,^^36^ The NLS1 173–183 is present from the Eukaryotic Linear Motif resource website (http://elm.eu.org/).^36^ The NLS2 is possibly amino acid residues 196–202 PIRKKSK and leads to nuclear localization of ELF4. The residue PIRKKSK is targeted at the nuclear pore complex by identifying importin-α.^37^^,^^38^ The function of residues PIRKKSK in ELF4 and PEQRKRK in ELF1 is analogous. In summary, amino acids 177–291 of ELF4 are necessary and enough for its nuclear localization.^33^ Interestingly, the retention of amino acids 177–291 in ELF4 results in more efficient nuclear localization than the retention of amino acids 177–206. This is because the longer sequence preserves the ETS domain, which in turn induces a conformational change in NLS2, enhancing its nuclear localization signal.^33^

## ELF4 as a transcriptional regulator

Transcription factors are a group of proteins that regulate gene transcription by binding to specific sequences of DNA.^39^ In particular, ELF4 plays a crucial role in the transcriptional regulation of important signaling pathways, such as the p53 pathway. Moreover, ELF4 interacts with various proteins to achieve coordinated regulation.

As a guardian of the genome, p53 reduces cell proliferation by inhibiting DNA damage, hypoxia, and nutrient deprivation, thereby attenuating tumorigenesis.^40^ However, its activity is frequently inactivated in most tumors.^41^^,^^42^ ELF4 acts as a transcriptional activator in the p53 pathway. Under normal physiological conditions, p53 is tightly regulated by E3 ligase mouse double minute 2 homolog (MDM2). MDM2 degrades p53 through the ubiquitin-proteasome system and p53 induces the expression of *Mdm2* by binding its promoter, which is a negative feedback loop to maintain the low levels of p53.^43^ Under cellular stress, the interaction between p53 and MDM2 is disrupted and p53 is activated.^44^ ELF4 activates *Mdm2* by binding to its promoter thereby repressing the stability of p53, which inhibits the transformation induced by p53-dependent oncogenic genes (such as *Ras* and *c-myc*) (Fig. 1B).^45^ In turn, p53 also regulates ELF4 stability. Likewise, activated p53 promotes the transcription of *MDM2* during DNA damage and then MDM2 affects the stability of ELF4 thereby inducing its degradation in the nuclear (Fig. 1B).^46^ Notably, MDM2 also reduces the stability of ELF4 in a p53-independent manner. ELF4/MDM2 axis and p53/MDM2 axis generate an autoregulatory negative feedback loop to keep the balance of cellular processes and specific roles depending on the cellular conditions. In addition, the transcriptional activator E2F1 (E2F transcription factor 1) facilitates *ELF4* expression by binding to its promotor, while p53 represses the ability of E2F1 to bind to DNA thereby blocking gene activation (Fig. 1C).^47^

The diverse function of ELF4 is also attributable to its binding partners (Fig. 1C). AML1 protein, also called runt-related transcription factor 1 (RUNX1), contains a highly conserved runt homology domain and plays a critical role in cellular proliferation and differentiation.^48^ ELF4 cooperates with the AML1 protein to activate the interleukin 3 (IL3) gene by interacting with runt homology domain in AML1 protein.^49^ Similarly, RUNX2 protein also has a runt domain.^50^ Therefore, ELF4 generates a complex with RUNX2 thereby suppressing its ability to bind to RUNX2-responsive elements on osteocalcin promoter DNA sequences.^51^ Recently, it has been demonstrated that ELF4 interacts with the N-terminal domain of nucleophosmin 1 (NPM1), and NPM1 represses the transactivation and DNA binding ability of ELF4 on the human *MDM2* promoter.^52^ Mutation *NPM1* reversals the effect on ELF4 and promotes the malignant transformation of ELF4-overexpressing NIH3T3 cells. ELF4 directly interacts with F-box protein 7 (FBXO7) as a strong activator.^53^ In addition, there is the negative feedback loop that FBXO7 represses the transactivation of ELF4 in an independent-ubiquitin ligase manner.

Other transcription factors also regulate the expression of ELF4 (Fig. 1C). In human epithelial cells, *ELF4* transcription is induced by Sp1, a ubiquitous zinc finger transcriptional activator by binding to its proximal 5′-flanking GC-rich promoter region.^54^ The transcriptional activity and function of ELF4 is enhanced under hypoxia conditions through HIF-1α (hypoxia-inducible factor 1 subunit alpha) binding to *ELF4* promoter (−200 bq).^55^

## Post-translational modifications of ELF4

Post-translational modifications contribute to the functional diversity of proteins thereby affecting various cellular functions.^56^^,^^57^ The ELF4 protein is modified by SUMOylation, phosphorylation, and ubiquitination, thereby playing a divergent role. Small ubiquitin-like modifiers (SUMOs) generate covalent bonds with specific lysine branches of target proteins including transcription factors ETS family, thereby down-regulating transcriptional activation.^17^^,^^58^ It has been demonstrated that lysine 657 of ELF4 is SUMO-modified in both *E. coli* and HEK293 cells, which down-regulates the transcriptional potency of ELF4 by reducing its recruitment to lysozyme promoter (Fig. 1A), but it does not influence cellular or subnuclear localization or stability of ELF4.^16^ The ELF4 mutation at lysine 657 to alanine leads to the abolition of SUMOylation, which clarifies the K657 is necessary and sufficient. However, the mechanism underlying the inhibition of SUMO-modified ELF4 recruitment to target promoters remains elusive. Phosphorylation is one of the most common post-translational modifications and is widely involved in regulating the activity of transcriptional factors.^59^ In osteoblasts, S641 of ELF4 is phosphorylated by parathyroid hormone-mediated c-Jun N-terminal kinase (JNK) thereby up-regulating the transcription of mab-21 like 1 (*MAB21L1*) (Fig. 1A).^60^ However, the ELF4 protein is phosphorylated by cyclin A-cyclin-dependent kinase 2 (CDK2) complex thereby decreasing DNA binding and inhibiting transcriptional activity.^15^ Specifically, the cyclin A-CDK2 complex targets three Ser/Thr residues within amino acids 641–657. Ubiquitin-proteasome degradation system increases the elimination of transcription factors.^59^ ELF4 protein is effectively degraded through a precise mechanism involving sequential phosphorylation events. Initially, ELF4 is phosphorylated by the cyclin D/cyclin dependent kinase 4 (CDK4) complex, followed by cyclin E/CDK2 or cyclin A/CDK2. This phosphorylation triggers the ubiquitination of ELF4, targeting it for degradation during the G1/S phase transition of the cell cycle.^61^ In the process, serine 648 of ELF4 is a key phosphorylation site for ubiquitination through cyclin A1/CDK2 (Fig. 1A). In addition, threonine 643 is the first phosphorylation site of ELF4 via cyclin E/CDK2.^61^ Furthermore, sequential phosphorylation-triggered SCF^Skp2^ complex regraded as an E3 ligase consisting of Skp1, Cul1, Rbx1 proteins, and Skp2 protein, mediates the attachment of ubiquitin molecules to phosphorylated ELF4 thereby inducing ubiquitination. Notably, the ELF4 S648A mutant is degraded through a mechanism that is not involved in ubiquitination, indicating that ELF4 degradation is not solely reliant on this process. It is reported that another E3 ligase MDM2 also mediates the ubiquitination and degradation of ELF4 independently of the phosphorylation of ELF4.^46^

## ELF4 in physiology

### Differentiation

Differentiation is a cornerstone process in the development of an organism, dictating the specialized characteristics and functions of various cell types. Transcription factors decide cell state and fate by interplaying with the three-dimensional genome.^62^ As an essential transcription factor, ELF4 is involved in the intricate regulatory networks that govern osteogenic, adipocyte, and neuronal differentiation.

#### Osteogenic differentiation

Osteogenic differentiation facilitates bone formation by enhancing mesenchymal stem cell differentiation into osteoblast and contributes to preventing osteoporosis.^63^ ELF4 is known to exert a suppressive impact on osteogenic differentiation. The expression of ELF4 is highest during the initial stage of differentiation in murine osteogenic cell line MC3T3-E1.^51^ Bone morphogenetic protein 2 (BMP-2) suppresses *Elf4* mRNA expression, and in turn, ELF4 opposes BMP-2 signaling. ELF4 suppresses bone formation by interacting with the osteoblast differentiation regulator RUNX2 protein and preventing RUNX2 from binding to osteoblast-specific elements (RUNX2 responsive element on osteocalcin promoter). ELF4 also inhibits the transcription of distal-less homeobox 5 (*Dlx5*), a BMP-2 effector for bone formation, and induces the transcription of msh homeobox 2 (*Msx2*), a negative regulator of osteoblast differentiation.^51^ *In vivo*, studies conducted on transgenic mice (*Col1α1-Elf4* Tg mice) show that overexpression of *Elf4* in osteoblasts reveals a correlation between increased osteoclast differentiation and several bone-related changes. These changes include osteopenia in the vertebrae, increased porosity in cortical bone, reduced trabeculation in long bones, elevated bone marrow adiposity, and a decrease in overall bone mass.^64^

#### Adipocyte differentiation

Peroxisome proliferator-activated receptor γ (PPARγ), a nuclear receptor, is a crucial mediator involved in lipid metabolism and inflammatory response.^65^ It has been observed that ELF4 enhances adipogenic differentiation by binding to the promoter of *Pparγ*, thereby transactivating its expression and intensifying its activity.^66^ PPARγ, in turn, drives adipocyte differentiation thereby enhancing bone loss and bone marrow adiposity.^67^ In addition, ELF4 overexpression induces MC3T3-E1 cells to secret more endogenous 15-Deoxy-Delta-12,14-prostaglandin J2 (15d-PGJ2) of the PPARγ ligand. Both osteoblasts and adipocytes are descended from mesenchymal stem cells. While ELF4 inhibits bone formation and osteoblast-associated factors, it simultaneously activates the expression of factors required for adipocyte differentiation. This dual role of ELF4 in cellular differentiation raises intriguing questions including its regulatory mechanisms, its impact on cell fate decisions, and its association with metabolic and bone disorders.^66^ ELF4 induces a transformation of mouse adipocytes into dermal fibroblast-like cells, presenting a novel therapeutic strategy for promoting the early closure of severe burn wounds.^68^

#### Neuronal differentiation

Lactate serves as the major glucose alternative to an energy substrate in the brain, and it is involved in brain development and neuronal differentiation.^69^^,^^70^ ELF4 is a specific transcription factor and is up-regulated by the lactate/N-Myc downstream-regulated 3 (NDRG3) axis in neuronal differentiation. ELF4 promotes the expression of neuronal marker genes such as synaptotagmin 4 (*SYT4*) in SH-SY5Y cells to facilitate neuronal differentiation.^70^

### Immune system

**Figure 2:**
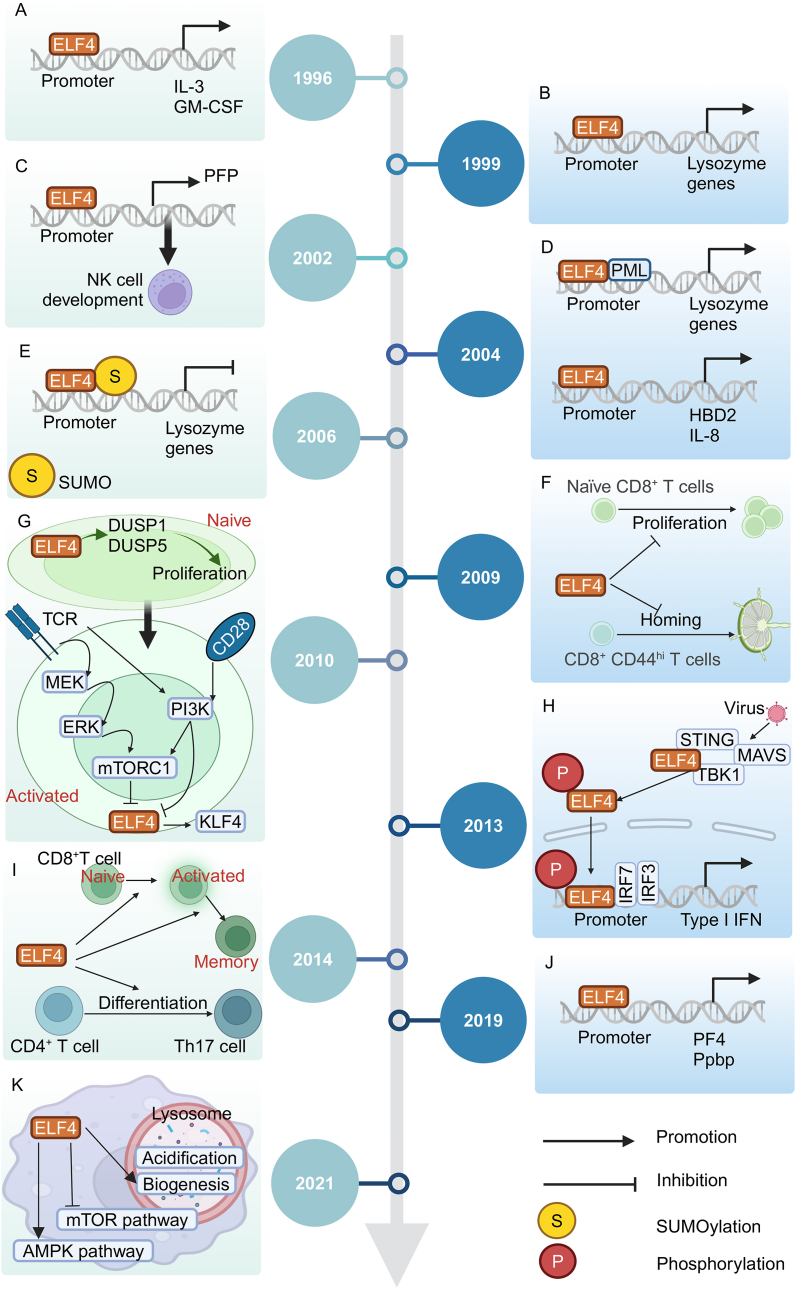
The timeline delineates the principal findings and pivotal advancements in ELF4-related immune system research so far. ELF4, E74 like ETS transcription factor 4; SUMO, small ubiquitin-like modifier; NK, natural killer; GM-CSF, granulocyte-macrophage colony-stimulating factor; IL-3/8, interleukin-3/8; PFP, perforin; HBD2, human β-defensin 2; SUMO, small ubiquitin-like modifier; DUSP1/5, dual-specificity phosphatase 1/5; TCR, T cell receptor; PI3K, phosphoinositide 3-kinase; KLF4, Krüppel-like factor 4; mTOR, mammalian target of rapamycin; mTORC1, mTOR complex 1; STING, stimulator of interferon gene; MAVS, mitochondrial antiviral signaling protein; TBK1, TANK-binding kinase 1; IRF3/7, interferon regulatory factor 3/7; IFN, interferon; PF4, platelet factor 4; Ppbp, pro-platelet basic protein; AMPK, AMP-activated protein kinase.

Growing evidence illustrates that ELF4 plays a crucial role in both innate and adaptive immune processes as a critical immune-related factor. Fig. 2 displays a timeline of the main findings and key advances in ELF4 research so far.

#### Immune molecules in innate immunity

ELF4 was first demonstrated to activate IL-3 and GM-CSF by specifically binding to their promoters, which are immune-related molecules in T and myeloid cell lines.^14^ Lysozyme is an important component of human innate immunity and plays an important role in mucosal defense. In the epithelial cell, ELF4 up-regulates the activity of the lysozyme 5A promoter and constitutively activates the expression of lysozyme genes.^71^ Additionally, the promyelocytic leukemia (PML) protein recruits ELF4 and promotes its accumulation in PML nuclear bodies. The interaction between PML and ELF4 enhances the transactivation of lysozyme genes, which is related to a proline-rich region of ELF4 in 447–517 amino acids.^72^ The effect is induced by heat shock. Notably, transcriptional activity of ELF4 on lysozyme genes is inhibited by SUMO modification.^16^ Antimicrobial peptide human β-defensin 2 is widely expressed in epithelial tissues and protects against microbial invasion by killing activity. ELF4 transactivates the expression of human β-defensin 2 by boosting the activity of binding to its promoter.^73^ Interestingly, PML protein acts similar function at the ELF4 regulation for β-defensin 2.^74^ It means that PML protein universally enhances the transactivation of ELF4. Interleukin-8 (IL-8) is a CXC chemokine and plays an essential role in attracting and activating neutrophils, angiogenesis, and immune response.^75^ In hematopoietic cells, ELF4 transactivates the expression of interleukin 8 (*IL8*) by strongly motivating its promoter.^76^ Type I interferon is a core and critical cytokine in the host antiviral defense response for innate immunity. After viral stimulation, ELF4 is recruited by stimulator of interferon genes (STING), interacts with the mitochondrial antiviral signaling protein (MAVS)-TANK-binding kinase 1 (TBK1) complex, and is activated through phosphorylation at Ser331, facilitating its nuclear import (Fig. 1A). Cooperating with interferon regulatory factor 3 (IRF3), IRF7, or p65, the activated ELF4 facilitates the expression of interferon gene by binding to the ELF4-IRF or ELF4-NF-κB composite motifs in the promoters of type I interferon.^36^ It was reported that homeodomain-interacting protein kinase 2 (HIPK2), a serine–threonine kinase, promotes the ELF4 phosphorylation at Ser369 thereby inducing DNA binding and transcription of interferon gene (Fig. 1A).^77^ Interestingly, after viral infection, ELF4 activates the expression of *Mir2*21 by binding to the GGAA region of its promoter.^78^ Next, up-regulation of miR-221 reduces interferon beta (IFN-β) and induces viral-infected impacts. Thus, the ELF4 regulation for interferon relies on a precise balance mechanism, which is expected to be further studied.

#### Immune cells in innate immunity

Natural killer (NK) cells and NK-T cells display a critical role in the earliest stage without prior sensitization of the immune response and kill the targeted cells by secreting cytotoxicity including perforin. ELF4 facilitates the expression of the perforin gene by directly binding to its promoter in NK cells.^18^ In the *Elf4*^*−*/−^ spleen, the number of NK cells is a 60% reduction and its function has an impairment of cytotoxicity and interferon-gamma (IFN-γ) secretion. In addition, the population of NK-T cells is a notable decline in the *Elf4*-deficient thymus and liver.^18^ ELF4 plays a distinctive role in shaping the development and functions of NK cells. This is further supported by a study conducted on patients who lack NK cells due to the p.T187N *ELF4* variant.^79^ There is a cross-talk between innate immunity and host anti-*plasmodium* defense. Human defense peptide platelet factor 4 (PF4) is expressed in megakaryocytes and secreted from platelets after *plasmodium* infection.^80^ ELF4 facilitates the transcription of *Pf4* and pro-platelet basic protein (*Ppbp*), thereby enhancing the PF4-induced killing of *plasmodium*-infected red cells.^81^ In macrophages, ELF4 enhances lysosome acidification and biogenesis, while inhibiting the mammalian target of rapamycin (mTOR) pathway by inducing AMP-activated protein kinase (AMPK) activity. Taken together, ELF4 facilitates *Staphylococcus aureus* clearance in macrophages and prevents tissue damage.^82^

#### Adaptive immunity

The balance of the T cell pool is maintained by a delicate regulation for quiescence and proliferation.^83^ In naïve CD8^+^ T cells, ELF4 induces Krüppel-like factor 4 (KLF4), a downstream signaling of T cell receptor (TCR), promoting cell cycle block and maintaining T cell quiescence.^84^ In *Elf4* or *Klf4*-deficient mice, naïve CD8^+^ T cells are activated for proliferation during quiescence or after immunization. In addition, CD8^+^ CD44^hi^ T cells are gradually increasing and are redistributed to nonlymphoid tissues with a reduction of KLF2, chemokine receptor 7 (CCR7), and L-selectin (CD62L) in *Elf4*^*−*/−^ mice.^84^ Hence, ELF4 represses the proliferation of naïve CD8^+^ T cells and regulates homing of CD8^+^ CD44^hi^ T cells. Furthermore, in resting naïve CD8^+^ T cells, ELF4 maintains the normal levels of dual-specificity phosphatase 1 (DUSP1) and DUSP5 to regulate the T cell proliferation after being activated and prevent weak stimulation.^85^ In activated CD8^+^ T cells, constant TCR/MEK/ERK and TCR/phosphoinositide 3-kinase (PI3K) stimulation down-regulates the expression of ELF4 and KLF4 by the mTOR pathway, thereby facilitating the proliferation of CD8^+^ T cells and immune effect. Co-stimulation of TCR with CD28 (cluster of differentiation 28) also leads to the repression of ELF4 independently of the mTOR pathway.^85^ Interestingly, under infection stimulation, ELF4 facilitates the activation and survival of naïve and effector CD8^+^ T cells.^86^ In addition, ELF4 induces the differentiation to effector memory CD8^+^ T cells and expands the recall response of effector memory CD8^+^ T cells and central memory CD8^+^ T cells partly by Notch1 signaling.^86^ However, while the cytotoxicity of CD8^+^ T cells is reduced, the numbers are not affected in *Elf4*^*−*/−^ spleen. To sum up, ELF4 plays a different role in resting and activating CD8^+^ T cells. Th17 cells have proinflammatory properties and are involved in mucosal immune responses. ELF4 indirectly suppresses the expression of interleukin 17A (IL17A) gene.^87^ In addition, ELF4 does not affect the proliferation and survival of naive CD4^+^ T cells, which serves as the precursor of Th17 cells, instead inhibits the commitment to the Th17 lineage differentiation by repressing the Notch1 signaling or reducing the transcriptional activity of Notch intracellular domain.^87^ To sum up, ELF4 represses the differentiation of CD4^+^ T cells into the Th17 lineage *in vivo* and *in vitro* and is a promising therapeutic target for autoimmune diseases.

## ELF4 in non-cancer diseases

### Immune-related diseases

Regarding the crucial roles of ELF4 in immune system development, its mutation results in immunodeficiency diseases. The clinical significance of ELF4 in the immune system is underscored by the evidence that the mutation in *ELF4* leads to X-linked hypogammaglobulinemia with growth hormone deficiency (XLH-GHD), which is a rare disease caused by immunodeficiency disorder.^88^ According to studies, patients with XLH-GHD carry a mutation at position 1487 from T to C, leading to an amino acid substitution from serine to proline at codon 369.^89^ Although serine 369 is not a vital phosphorylation location in various research,^15^ the study speculated that the mutation at this site may lead to interference with the capability of ELF4 to interact with other proteins or its capacity to attach to DNA. Alternatively, the mutation affects the precise folding of ELF4 protein as well. Additionally, patients who suffer from XLH-GHD present diverse symptoms, such as chronic infections, tissue inflammations, and arthritis. Moreover, these patients show similar clinical features to individuals suffering from the more regular X-linked agammaglobulinemia, which is a result of mutations in the Bruton's tyrosine kinase (*BTK*) gene.^90^ Additionally, research has indicated a correlation between the severity of common variable immunodeficiency, like hypogammaglobulinemia, and NK cell count. Patients with common variable immunodeficiency and lower NK cell counts tend to have more severe disease manifestations.^91^

### Inflammatory-related diseases

Lots of bioinformatic analyses display that ELF4 is abundant in multiple inflammation-related diseases including multiple sclerosis and systemic lupus erythematosus.^92^^,^^93^ Sun et al described a pediatric patient with a hemizygous variant in *ELF4*, demonstrating that mutation results in reduced expression of antiviral- and anti-inflammation-associated genes due to the impaired ability of mutant *ELF4* to bind to the promoters of these genes.^94^ Additional patients are reported and their main clinical manifestations are oral ulcer, inflammatory bowel disease-like symptoms, fever of unknown origin, anemia, or systemic lupus erythematosus. Whole exome sequencing revealed that all cases acquired potential pathogenic variants in *ELF4*.^95^ Loss-of-function variants in *ELF4* lead to early-onset mucosal autoinflammation and inflammatory bowel disease features due to hyperinflammatory responses of macrophages to innate stimuli. ELF4 maintains the expression of anti-inflammatory genes and limits the induction of inflammation amplifiers. Thus, ELF4 attenuates inflammation and protects against mucosal diseases, which provides a potential targeted therapeutic strategy for human inflammatory diseases including inflammatory bowel disease.^96^ Intestinal ELF4 plays a crucial role in sustaining gut homeostasis and protecting against alcohol-induced liver injury. Its deficiency leads to gut dysbiosis and dysfunction of the intestinal barrier, leading to exacerbated liver steatosis and inflammation.^97^ The down-regulation of ELF4 elevates ischemia/reperfusion injury thereby aggravating acute kidney injury. The precise mechanism is possibly associated with oxidative stress, endoplasmic reticulum stress, and inflammation.^98^ Thus, further research on targeting ELF4 provides a novel therapeutic choice for inflammation-related diseases.

## ELF4 in cancer

### Dysregulation of ELF4

**Figure 3:**
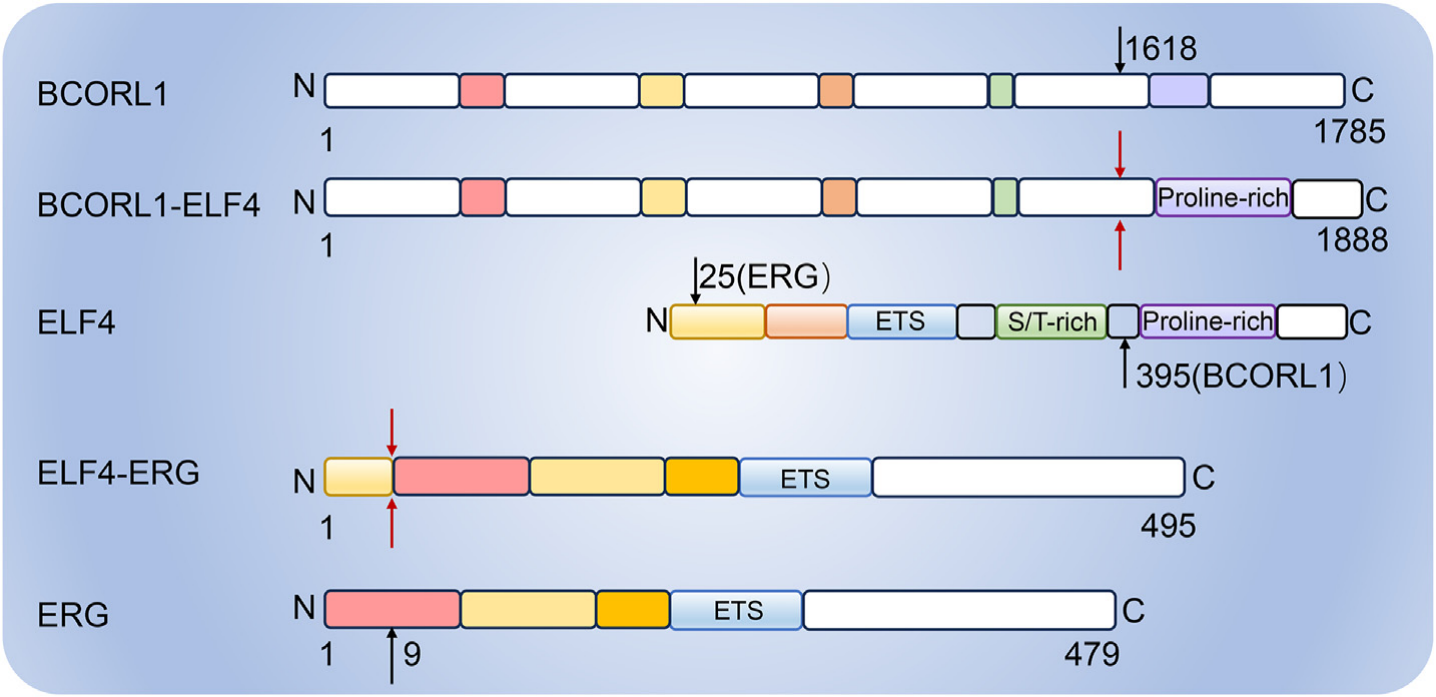
The protein fusion between BCORL1 and ELF4 or ERG and ELF. In BCORL1-ELF4 fusion, the breakpoint site of BCORL1 is reported at 1618 amino acids and that of ELF4 is at 395 amino acids. In ELF4-ERG fusion, the breakpoint site of ERG is reported at 9 amino acids and that of ELF4 is at 25 amino acids. ELF4, E74 like ETS transcription factor 4; ERG, ETS transcription factor; BCORL1, BCL6 corepressor like 1.

ELF4 exerts an essential impact on the regulation of physiological function. However, some adverse signaling stimulates the dysregulation of ELF4 thereby inducing pathological lesions and even tumorigenesis. ELF4 acts as a tumor suppressor and is inactivated in multiple cancers.^20^ As mentioned above, AML1 interacts with ELF4 and plays a critical role in the development of hematopoiesis.^49^ However, in acute myeloid leukemia (AML), t (8; 21)-induced AML1/ETO fusion protein abolishes the interaction with ELF4.^49^ In turn, the transcription activity of ELF4 is inhibited and its dysregulation promotes AML progression by disrupting the myeloid differentiation.^49^ In acute promyelocytic leukemia, promyelocytic leukemia (PML)/retinoic acid receptor alpha (RARα) and promyelocytic zinc finger (PLZF)/RARα fusions down-regulate the expression of ELF4.^99^ The dysregulation of ELF4 has an essential influence on the two types of AML. Additionally, one case report has shown that the t (X; 21) (q25-26; q22) in AML drives the fusion between *ELF4* and *ERG* (ETS transcription factor), indicating the involvement of ELF4 in cancer (Fig. 3).^100^ In turn, the expression of *ELF4/ERG* fusion results in transcriptome disorder, which is expected to be studied to clarify pathogenesis and functional mechanisms.

Although lots of studies have reported that ELF4 is inhibited or deleted to facilitate tumorigenesis as a tumor suppressor, it also acts pro-oncogenic function in some tumors. ELF4 is highly expressed in various cancers, including leukemia, ovarian cancer, liver cancer, papillary thyroid cancer, and nasal NK/T-cell lymphomas.^19^^,^^101^, ^102^, ^103^, ^104^ In liver cancer, a BCL6 corepressor like 1 (*BCORL1*)*-ELF4* fusion has been identified, which causes a more than six-fold increase in the transcription of the fusion gene than the expression of wide-type *BCORL1* and *ELF4* gene in non-cancerous liver tissues (Fig. 3).^101^ Additionally, in murine cancer models, ELF4 is activated and serves as an inserted site for retroviral mutagenesis.^105^^,^^106^ Thus, it is crucial to illustrate the context-dependent roles of ELF4 in cancer. Table 1 shows the regulation and functions of ELF4 in various cancers.

**Table 1:** The role of ELF4 in cancer.

Cancer	Expression of ELF4	Molecular mechanism	Effect	Clinical significance	Reference
AML	Down-regulation	AML1/ETO fusion protein disturbs the interaction with ELF4	–	Associated with good prognosis	49
APL	Down-regulation	PML/RARα and PLZF/RARα fusions	–	–	99
NKTCL-N	Up-regulation	–	–	A diagnostic biomarker	102
NSCLC	Down-regulation	Inhibits the expression of *MMP9* and *IL8*	Inhibits growth and invasion	–	20
OC	Up-regulation	–	Enhances the growth properties	–	19
HCC	–	Activates TERT expression	Boosts sphere-developed ability of HCC cells	–	109
GC	Up-regulation	Be induced by lncRNA LINC01091 binding to miR-128-3p, activates CDX2 expression	Facilitates growth, migration, invasion, and metastasis	–	111
CRC	Up-regulation	Be up-regulated by FGF19/ERK1/2/SP1, activates FGFR4 and SRC	Induces metastasis	Associated with distant metastasis, advanced AJCC stages, and poor outcomes, an independent biomarker for poor prognosis	22
Glioma	Up-regulation	Be up-regulated by lncRNA PVT1 sponging miR-365, promotes SOX2 expression	Enhances CSC properties and promotes tumor occurrence	Temozolomide resistance, associated with short survival times	21,114
EC	Up-regulation	Be recruited to the promoter of *CTNNB1* (encoding β-catenin) by TRIB3	Enhances oncogenesis and self-renewal of CSCs	–	115
OSCC	Down-regulation	Missense mutation (L211M) of ELF4, reduces HRK and DLX3	Inhibits proliferation	–	125
NB	Up-regulation	Influences DREAM complex as a target of miR-124	Promotes proliferation	Associated with poor survival	126
GBM	Up-regulation	Regulate RTK signaling-associated (SRC, PTK2B and TNK2) and lipid dynamics-associated (LRP1, APOE, ABCA7, PLA2G6 and PITPNM2) genes	Promotes proliferation	Associated with short survival times	21,129,131
ESCC	Up-regulation	Facilitate the transcription of FUT9 gene	Enhances cancer stem-like properties and promotes proliferation, invasion, and migration	Associated with poor prognosis and tumor stages	116

Note: ELF4, E74 like ETS transcription factor 4; AML, acute myeloid leukemia; APL, acute promyelocytic leukemia; NKTCL-N, NK/T-cell lymphomas-nasal type; NSCLC, non-small cell lung carcinoma; OC, ovarian cancer; HCC, hepatocellular carcinoma; GC, gastric cancer; CRC, colorectal cancer; EC, endometrial cancer; OSCC, oral squamous cell carcinoma; NB, neuroblastoma; GBM, glioblastoma; ESCC, esophageal squamous cell carcinoma; FGF19, fibroblast growth factor 19; CTNNB1, catenin beta 1; FUT9, fucosyltransferase 9; SRC, non-receptor tyrosine kinase; PTK2B, protein tyrosine kinase 2 beta; TNK2, tyrosine kinase non-receptor 2; LRP1, LDL receptor related protein 1; APOE, apolipoprotein E; ABCA7, ATP binding cassette subfamily A member 7; PLA2G6, phospholipase A2 group VI; PITPNM2, phosphatidylinositol transfer protein membrane associated 2; HRK, Harakiri, BCL2 interacting protein; DLX3, distal-less homeobox 3; TRIB3, tribbles pseudokinase 3; FGFR4, fibroblast growth factor receptor 4; CDX2, caudal type homeobox 2; MMP9, matrix metallopeptidase 9; IL8, interleukin 8; PML, promyelocytic leukemia; RARα, retinoic acid receptor alpha; PLZF, promyelocytic zinc finger; TERT, telomerase reverse transcriptase; SOX2, SRY-box transcription factor 2.

### Invasion and metastasis

Invasion and metastasis are the primary causes of mortality in cancer patients.^107^ ELF4 plays a bidirectional role in tumor suppression and tumorigenesis, with its role being dependent on cellular context. As a potential tumor suppressor, ELF4 is down-regulated by methylation in multiple cancers. ELF4 facilitates DNA damage repair by inducing the transcription of DNA damage repair genes, which is activated by PARylation of poly (ADP-ribose) polymerases 1 (PARP1).^108^ Additionally, ELF4 suppression mediated by DNA methyltransferase 1 (DNMT1) methylation of its promoter drives the transformation of ulcerative colitis to colitis-associated cancer.^108^ In human non-small cell lung carcinoma A549 cells, ELF4 inhibits the expression of matrix metallopeptidase 9 (*MMP9*) and *IL8* by reducing their promoter activities, thereby repressing tumor growth and invasiveness.^20^ However, there is no solid evidence of *ELF4* acting as a tumor suppressor gene and the *ELF4* mutations have not yet been found in human cancers. *Elf4*-deficient mice do not spontaneously develop tumors even at old age.^18^ In contrast, ELF4 promotes the growth properties of ovarian cancer cell lines (SKOV3 cells and CAOV3 cells) and induces the malignant transformation of NIH3T3 cells.^19^ Telomerase reverse transcriptase (TERT) is activated to drive the occurrence of hepatocellular carcinoma as an inserted target of hepatitis B virus in hepatitis B virus-associated hepatocellular carcinoma.^109^ It is demonstrated that ELF4 plays a critical role in TERT activation. The knockdown of ELF4 inhibits the expression of TERT and reduces the sphere-forming ability of hepatocellular carcinoma cells.^110^ In gastric cancer, exosomal lncRNA LINC01091 enhances the expression of ELF4 by binding to miR-128-3p. In turn, ELF4 transactivates the expression of caudal type homeobox 2 (CDX2), thereby boosting growth, migration, invasion, and metastasis of gastric cancer.^111^ In colorectal cancer, overexpression of ELF4 transactivates fibroblast growth factor receptor 4 (FGFR4) and non-receptor tyrosine kinase (SRC) to facilitate the metastasis of colorectal cancer.^22^ Furthermore, fibroblast growth factor 19 (FGF19) up-regulates the expression of ELF4 by the ERK1/2/SP1 axis as a specific ligand of FGFR4. Thus, FGF19/ELF4/FGFR4 generates a positive feedback loop to enhance colorectal cancer cell metastasis.

### ELF4 in stemness

Cancer stem cells are identified as a group of cells within cancers that possess the ability to self-renew and contribute to the initiation, progression, metastasis, and recurrence of cancers.^112^^,^^113^ In glioma, high ELF4 expression up-regulates the SRY-box transcription factor 2 (SOX2) expression, enhancing cancer stem cells' features and promoting tumor occurrence.^21^ In addition, lncRNA PVT1 sponges miR-365 to up-regulate ELF4, which in turn serves as an upstream regulator of SOX2, thereby facilitating the stemness features and temozolomide resistance of glioma.^114^ In endometrial cancer, ELF4, acting as a transcriptional activator, is recruited to the promoter of catenin beta 1 (*CTNNB1*; encoding β-catenin) by tribbles pseudokinase 3 (TRIB3), thereby enhancing oncogenesis and self-renewal of cancer stem cells.^115^ Similarly, ELF4 is up-regulated and facilitates the expression of fucosyltransferase 9 (*FUT9*; encoding a cancer stem-like properties affecter) in esophageal squamous cell carcinoma, thereby enhancing cancer stem-like properties and promoting tumor progression.^116^

### ELF4 in tumor microenvironment

Tumor microenvironment (TME) is a complex system containing tumor cells, various immune cells, and other matrix components.^117^ ELF4 has been demonstrated to play a critical role in physiological immune defense. Thus, the function of ELF4 in tumor immunity is a subject that needs clarification. Recently, it has been reported that ELF4 is also involved in infiltrating immune cells in the TME. The expression of ELF4 is significantly associated with immune cell infiltration (CD4^+^ cells, CD8^+^ cells, and neutrophils) and immune genes (positively in *CD163*, *CD22*, *CD27*, *CD33*, *CD4*, *CD80*, *CD86*, forkhead box P3 (*FOXP3*), and Toll-like receptor 2 (*TLR2*), while negatively in *CD24*).^118^ In macrophages, exosomal circZNF451 facilitates the ubiquitination of RNA-binding protein FXR1 (FMR1 autosomal homolog 1) by E3 ligase TRIM56, thereby activating the ELF4-interferon regulatory factor 4 (IRF4) pathway.^119^ The circZNF451/FXR1/ELF4/IRF4 axis reshapes the tumor immune microenvironment by inducing the polarization of M2 macrophages, which subsequently inhibits the sensitivity of anti-programmed death-1 (PD-1) therapy in lung adenocarcinoma. Notably, the conditional knockdown of ELF4 in macrophages rescues the therapeutic effect of anti-PD-1 treatment.^119^

**Figure 4:**
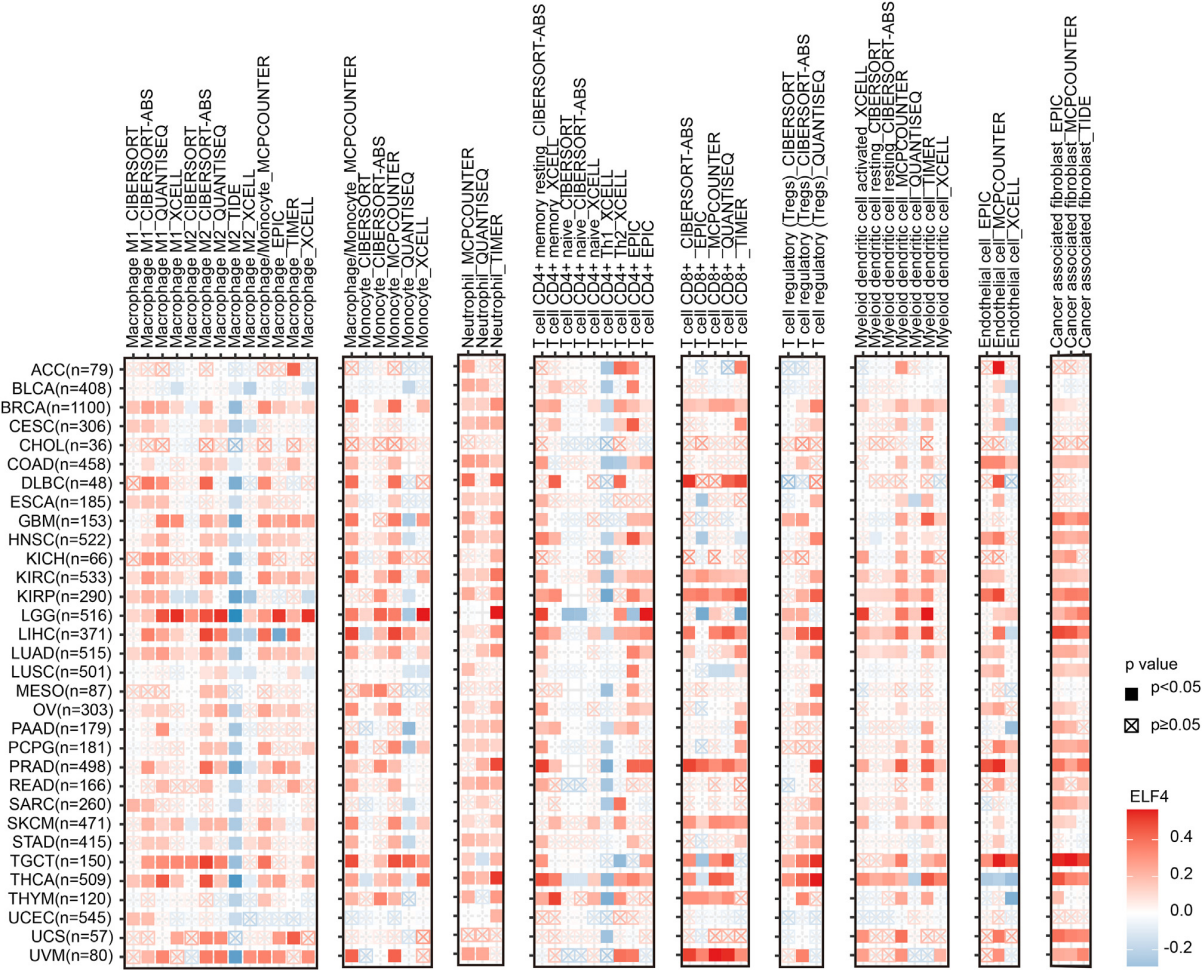
The heatmap shows the correlation between ELF4 expression and the abundance of tumor-infiltrating immune cells in multiple tumors using the TIMER 2.0 database. ELF4, E74 like ETS transcription factor 4; ACC, adrenocortical carcinoma; BLCA, bladder urothelial carcinoma; BRCA, breast invasive carcinoma; CESC, cervical squamous cell carcinoma; CHOL, cholangiocarcinoma; COAD, colon adenocarcinoma; DLBC, diffuse large B-cell lymphoma; ESCA, esophageal carcinoma; GBM, glioblastoma; HNSC, head and neck squamous cell carcinoma; KICH, kidney chromophobe; KIRC, kidney clear cell carcinoma; KIRP, kidney renal papillary cell carcinoma; LGG, lower grade glioma; LIHC, liver hepatocellular carcinoma; LUAD, lung adenocarcinoma; LUSC, lung squamous cell carcinoma; MESO, mesothelioma; OV, ovarian cancer; PAAD, pancreatic adenocarcinoma; PCPG, pheochromocytoma and paraganglioma; PRAD, prostate adenocarcinoma; READ, rectal adenocarcinoma; SARC, sarcoma; SKCM, skin cutaneous melanoma; STAD, stomach adenocarcinoma; TGCT, testicular germ cell tumor; THCA, thyroid carcinoma; THYM, thymoma; UCEC, uterine corpus endometrioid cancer; UCS, uterine carcinosarcoma; UVM, ocular melanomas.

To further explore the ELF4 potential in regulating tumor immune microenvironment, we deeply examine the correlation between ELF4 expression and various tumor-infiltrating immune cells across different tumor types using the Tumor Immune Estimation Resource (TIMER) 2.0 database analysis (Fig. 4). In most tumors, ELF4 expression has a positive correlation with macrophages (M1 and M2), monocytes, neutrophils, CD4^+^ T cells, CD8^+^ T cells, regulatory T cells, myeloid dendritic cells, endothelial cells, and cancer-associated fibroblasts. The results suggest that ELF4 may exert crucial functions in regulating both innate and adapt immunocytes in the TME. Furthermore, the association with regulatory T cells suggests that ELF4 may be involved in the balance of immune responses and immune tolerance as a key transcription factor. The correlation of ELF4 expression with endothelial cells and cancer-associated fibroblasts also indicates the potential roles of ELF4 in regulating angiogenesis and tumor extracellular matrix remodeling in the TME. It is meaningful to explore the regulatory roles and mechanisms of ELF4 in various immune cells of the TME.

**Figure 5:**
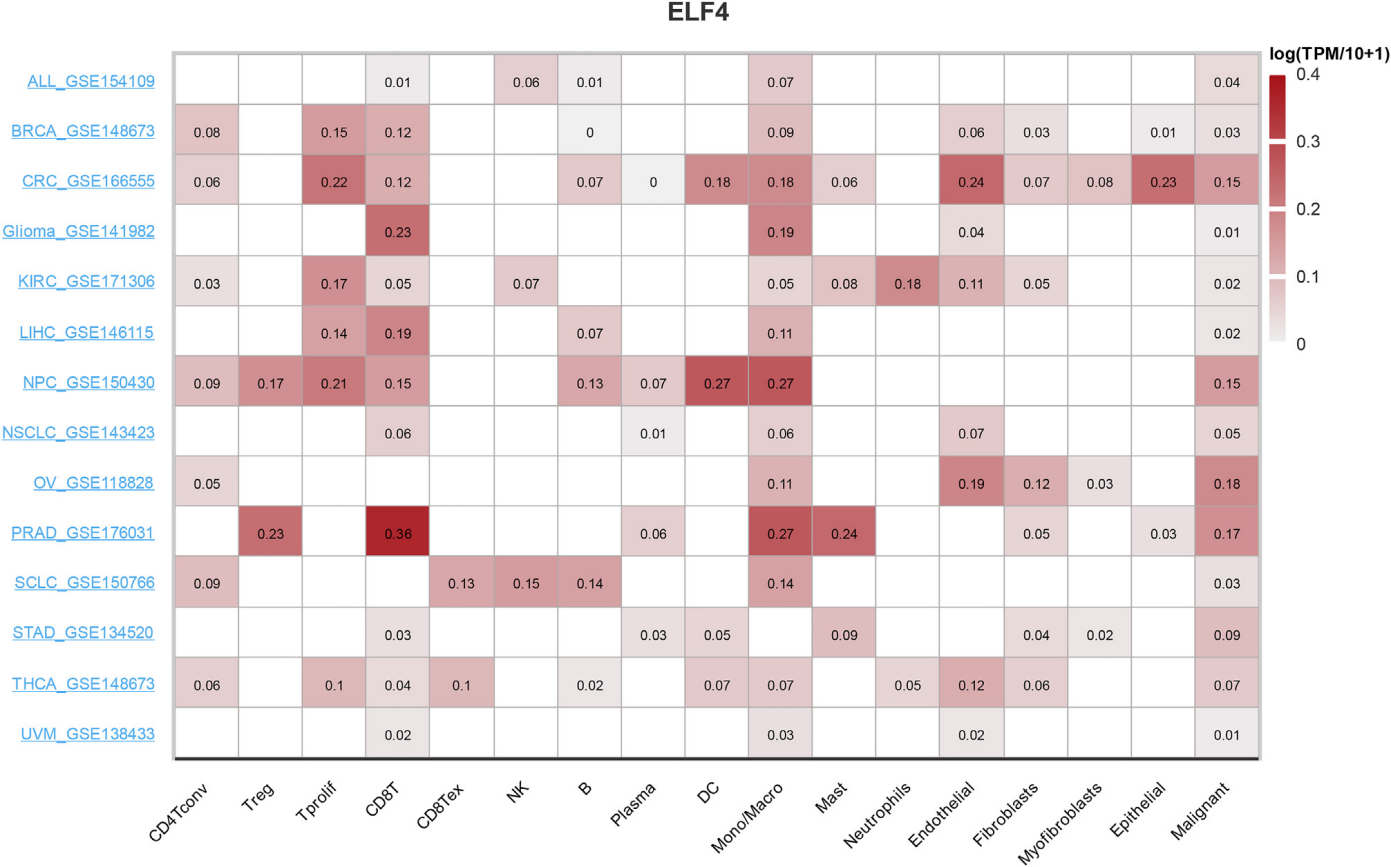
The heatmap displays the correlation between ELF4 expression and tumor microenvironment at the single-cell level using the TISCH database. ELF4, E74 like ETS transcription factor 4; ALL, acute lymphoblastic leukemia; BRCA, breast invasive carcinoma; CRC, colorectal cancer; KIRC, kidney renal clear cell carcinoma; LIHC, liver hepatocellular carcinoma; NPC, nasopharyngeal carcinoma; NSCLC, non-small cell lung carcinoma; OV, ovarian cancer; PRAD, prostate adenocarcinoma; SCLC, small cell lung cancer; STAD, stomach adenocarcinoma; THCA, thyroid carcinoma; UVM, ocular melanomas.

Furthermore, we analyzed the correlation between ELF4 expression and the TME at the single-cell level using the Tumor Immune Single-cell Hub (TISCH) database (Fig. 5). In colorectal cancer, ELF4 expression is significantly positively associated with proliferating T (Tprolif) cells, endothelial cells, and epithelial cells. In nasopharyngeal carcinoma, ELF4 expression shows a significant positive association with Tprolif cells, dendritic cells, and monocytes/macrophages. In prostate adenocarcinoma, ELF4 expression is significantly positively associated with regulatory T cells, CD8^+^ T cells, monocytes/macrophages, and mast cells. Importantly, across all analyzed cancers, ELF4 expression is positively correlated with malignant cells, which hints that ELF4 may affect tumor malignant features positively. From a cellular perspective, ELF4 expression is mainly associated with CD8^+^ T cells, monocytes/macrophages, and malignant cells in most cancer types. These results further indicate that ELF4 plays a role in immune surveillance and the killing effects of immunocytes. In summary, the association between ELF4 expression and multiple cell types in the TME is complex, and the ELF4 function in the TME is extremely potent.

### Proliferation

Cancer cells have the characteristic to sustain proliferation and even immortality.^120^ ELF4 is involved in the proliferation regulation in multiple cancers. ELF4 promotes the cell cycle-entry of hematopoietic stem cells (HSCs).^121^ At steady state, the down-regulation or deficiency of ELF4 maintains the quiescence of primary hematopoietic progenitors (long-term HSCs and short-term HSCs) and enhances the abundance of HSCs instead of retention in bone marrow. These commonly contribute to hematopoietic recovery after chemotherapy or radiotherapy.^121^ In certain subtypes of AML, the down-regulation of ELF4 expression possibly promotes AML pathogenesis and resistance to chemotherapy by altering the growth properties of leukemic stem cells, similar to what is observed in *ELF4*-deficient HSCs. Deficiency of ELF4 also regulates the quiescence of human umbilical vein endothelial cells by down-regulating CDK4 expression.^122^ ELF4 suppresses the roles of p53 in inducing HSC quiescence while being independent of p53 in inhibiting HSC self-renewal.^123^^,^^124^ In oral squamous cell carcinoma cells, a missense mutation (L211M) in ELF4 leads to suppressed transactivation. In turn, the inactivation of ELF4 disrupts the antiproliferative effects by reducing HRK (Harakiri, BCL2 interacting protein; an apoptotic activator) and DLX3 (distal-less homeobox 3; a growth inhibitory factor).^125^ Furthermore, ELF4 exhibits pro-proliferative effects in cancer. In neuroblastoma, ELF4 facilitates the proliferation of cancer cells by affecting the dimerization partner, RB-like, E2F, and multi-vulval class B (DREAM) complex and maintains an undifferentiated state of the tumor, acting as a target of miR-124.^126^ The RTK signaling mediates the proliferation by triggering a cascade response and is affected by lipid dynamics.^127^^,^^128^ In glioblastoma, ELF4 regulates the expression of genes associated with both receptor tyrosine kinase (RTK) signaling (SRC, protein tyrosine kinase 2 beta (PTK2B), and tyrosine kinase non-receptor 2 (TNK2)) and lipid dynamics (LDL receptor-related protein 1 (LRP1), apolipoprotein E (APOE), ATP binding cassette subfamily A member 7 (ABCA7), phospholipase A2 group VI (PLA2G6), and phosphatidylinositol transfer protein membrane-associated 2 (PITPNM2)), thereby promoting cancer cell proliferation.^129^

### ELF4 as a prognosis biomarker

Gene expression profiles provide a basis for disease diagnosis and prognosis. In M2 and M3 AML, low relative expression of ELF4 is associated with good prognosis, which is expected to be further investigated with a broader range of samples.^130^ ELF4 is highly expressed in neuroblastoma and is positively correlated with poor patient survival.^126^ In both human and mouse glioblastoma, ELF4 is highly expressed and patients with lower expression of ELF4 tend to have longer survival times.^21^^,^^131^ Based on bioinformatics analysis, cancers with elevated ELF4 expression are correlated with a higher grade, older patients, and a greater mutation burden. Furthermore, patients with ELF4 overexpression often have worse outcomes and wider drug resistance.^23^ In HuCCT1 cells, the expression of ELF4 is up-regulated and ELF4 is a potential prognostic biomarker for cholangiocarcinoma.^118^ In colorectal cancer, increased ELF4 expression is positively associated with distant metastasis, advanced American Joint Committee on Cancer (AJCC) stages, and poor patient outcomes.^22^ In esophageal squamous cell carcinoma, ELF4 is associated with poor prognosis and advanced tumor stages. Patients with lower ELF4 levels have better overall survival.^116^ ELF4 is an independent biomarker for predicting poor prognosis in colorectal cancer. Thus, clarifying the role of ELF4 contributes to evaluating patient prognosis and identifying effective therapeutic targets.

### Perspectives

Table 2 and Table 3 illustrate that ELF4 is a core transcription factor in the regulatory network, involved in various biological processes. Although extensive studies have discussed the roles of ELF4, there remain some unanswered questions regarding its concrete roles and mechanisms.

**Table 2:** The downstream targets of ELF4.

Target	Effect	Role	Reference
MDM2	Promotion	Promotes the ubiquitination degradation of p53	45
DLX5	Inhibition	Suppresses the bone formation	51
MSX2	Promotion	Inhibits osteogenic differentiation	51
PPARγ	Promotion	Facilitates adipogenic differentiation by binding to the promoter of Pparγ	66
SYT4	Promotion	Facilitates the neuronal differentiation	70
Perforin	Promotion	Maintains the function of natural killer cells	18
PF4	Promotion	Induces killing for *plasmodium*-infected red cells	81
Ppbp			
MMP9	Inhibition	Attenuates tumor growth and invasiveness in human non-small cell lung carcinoma A549 cells	20
TERT	Promotion	Enhances the sphere-developed ability of hepatocellular carcinoma cells	110
FGFR4	Promotion	Facilitates the metastasis of colorectal cancer	22
SRC			
SOX2	Promotion	Promotes the cancer stem cells' properties and oncogenesis	21
CTNNB1	Promotion	Boosts oncogenesis and self-renewal of cancer stem cells in endometrial cancer	115
FUT9	Promotion	Enhances cancer stem-like properties and tumor progression in esophageal squamous cell carcinoma	116
CDK4	Inhibition	Regulates the quiescence of human umbilical vein endothelial cells	122

Note: ELF4, E74 like ETS transcription factor 4; CTNNB1, catenin beta 1; DLX5, distal-less homeobox 5; FUT9, fucosyltransferase 9; CDK4, cyclin dependent kinase 4; MSX2, msh homeobox 2; MDM2, mouse double minute 2 homolog; PF4, platelet factor 4; Ppbp, pro-platelet basic protein; FGFR4, fibroblast growth factor receptor 4; PPARγ, peroxisome proliferator-activated receptor γ; SYT4, synaptotagmin 4; MMP9, matrix metallopeptidase 9; TERT, telomerase reverse transcriptase; SRC, non-receptor tyrosine kinase; SOX2*,* SRY-box transcription factor 2.

**Table 3:** The upstream regulation of ELF4.

Upstream regulator	Effect on ELF4	Role	Reference
P53	Inhibition	Promotes the ubiquitination degradation of ELF4 by up-regulating MDM2	46
E2F1	Promotion	Promotes the expression of ELF4 by binding the E2F consensus site of its promoter	47
FBXO7	Inhibition	Suppresses the transcription activity of ELF4 in an independent-ubiquitin ligase manner	53
Sp1	Promotion	Facilitates the transcription of ELF4 by binding to its proximal 5′-flanking GC-rich promoter region	54
HIF-1α	Promotion	Enhances the transcription activity of ELF4 by binding to its promoter	55
BMP-2	Inhibition	Regulates the osteogenic differentiation	51
Lactate/NDRG3	Promotion	Facilitates the neuronal differentiation	70
TCR/MEK/ERK	Inhibition	Enhances the proliferation of CD8^+^ T cells	85
TCR/PI3K			
DNMT1	Inhibition	Drives the malignant transformation of ulcerative colitis	108
lncRNA LINC01091/miR-128-3p	Promotion	Promotes growth, migration, invasion, and metastasis of gastric cancer	111
FGF19/ERK1/2/SP1	Promotion	Forms a FGF19/ELF4/FGFR4 feedback loop to enhance colorectal cancer cell metastasis	22
lncRNA PVT1/miR-365	Promotion	Facilitates the stemness feature and temozolomide resistance of glioma	114

Note: ELF4, E74 like ETS transcription factor 4; FGF19, fibroblast growth factor 19; MDM2, mouse double minute 2 homolog; FBXO7, F-box protein 7; HIF-1α, hypoxia inducible factor 1 subunit alpha; E2F1, E2F transcription factor 1; TCR, T cell receptor; PI3K, phosphoinositide 3-kinase; FGFR4, fibroblast growth factor receptor 4; BMP-2, bone morphogenetic protein 2; NDRG3, N-Myc downstream-regulated 3; DNMT1, DNA methyltransferase 1.

As mentioned above, ELF4 plays a divergent role in cancer. ELF4 is down-regulated to promote tumor progression in some cancers, while ELF4 is highly expressed in other cancers and facilitates tumorigenesis. Tumor heterogeneity may contribute to the differing expression and effects of ELF4. Hence, it is necessary to map the panorama of ELF4 in tumors. However, most of the molecular mechanisms of ELF4 in cancer are not yet clarified. Understanding its regulation could provide the potential for precision medicine and individualized treatment.

Research on ELF4 in immune system development is ongoing comprehensive and in-depth. The deficiency or mutation of ELF4 contributes to the occurrence of immune-related diseases. Additionally, the association between ELF4 expression and multiple cell types including immune-infiltering cells in the TME, reveals that ELF4 may play a key role in complex network regulation of the TME. Its precise mechanisms in the TME remain unclear. Given its essential role in immunity, ELF4 is expected to serve as an immune therapeutic target. There is no doubt that further research on ELF4 in physiological immunity, pathological immunity, or tumor immunity is an inspiring and promising direction.

ELF4 is critical for zygotic gene activation and epigenetic reprogramming during early embryonic development in pigs.^132^ However, whether it has a similar effect in humans has not been studied. Further research on clarifying the ELF4 regulation in human embryonic development could contribute to early intervention for diseases mediated by mutant ELF4.

## Conclusion

Genomic and biochemical research has demonstrated that ELF4 is a critical transcriptional factor in regulating physiological cellular behavior and plays a dual regulatory role in cancer. However, ELF4 inhibitors have yet to be discovered and the therapeutic strategies that target the upstream and downstream pathways of ELF4 are expected to be further explored. Growing clinical studies have shown that ELF4 is significantly correlated with poor prognosis in cancer, suggesting its potential for early diagnoses and prognosis assessments. Thus, clarification of the ELF4-mediated molecular mechanisms and the development of corresponding strategies will certainly contribute to transforming basic research into clinical practice.

## Funding

This research was funded by the 10.13039/501100001809National Natural Science Foundation of China (No. U23A20451, 82273310, 82372917, 82173313), the 10.13039/501100003819Natural Science Foundation of Hubei Province, China (No. 2022CFA016), and the Basic Research Support Program of 10.13039/501100003397Huazhong University of Science and Technology (China) (No. 2023BR038).

## Data availability

The datasets generated and/or analyzed during the current study are available in the TIMER 2.0 database (http://timer.cistrome.org/) and the TISCH database (http://tisch.comp-genomics.org/home/).

## CRediT authorship contribution statement

**Dian Hu:** Conceptualization, Investigation, Methodology, Writing – original draft. **Zerui Zhang:** Data curation, Software, Writing – original draft. **Yijun Wang:** Investigation, Visualization, Writing – original draft. **Siwen Li:** Methodology, Visualization, Writing – original draft. **Jiaqian Zhang:** Investigation, Visualization. **Zhangfan Wu:** Investigation, Methodology. **Mengyu Sun:** Writing – review & editing. **Junqing Jiang:** Writing – review & editing. **Danfei Liu:** Writing – review & editing. **Xiaoyu Ji:** Writing – review & editing. **Shuai Wang:** Supervision, Writing – review & editing. **Yufei Wang:** Supervision, Validation, Writing – review & editing. **Xiangyuan Luo:** Conceptualization, Resources, Supervision, Writing – review & editing. **Wenjie Huang:** Funding acquisition, Validation, Writing – review & editing. **Limin Xia:** Conceptualization, Funding acquisition, Resources, Supervision, Writing – review & editing.

## Conflict of interests

The authors declared no competing interests.
